# A Relationship Between Atrial Fibrillation and Dizziness

**DOI:** 10.3390/jcm14197101

**Published:** 2025-10-09

**Authors:** Rodica Urs, Luigi Geo Marceanu, Horatiu Rus, Alexandru Covaciu, Christian Gabriel Strempel, Elena Bobescu

**Affiliations:** 1 Clinical Children’s Hospital Brasov, 500063 Brasov, Romania; rodyk_16@yahoo.com; 2 Department of Medical and Surgical Specialties, Faculty of Medicine, Transilvania University of Brasov, 500036 Brasov, Romaniaelena.bobescu@gmail.com (E.B.); 3 Department of Cardiology, Clinical County Emergency Hospital Brasov, 500059 Brasov, Romania; 4 Department of Finance, Accounting and Economic Theory, Faculty of Economic Sciences and Business Administration, Transilvania University of Brasov, 500036 Brasov, Romania

**Keywords:** vertigo, dizziness, atrial fibrillation

## Abstract

**Background**: Vertigo and dizziness have been observed in patients with atrial fibrillation (AF), yet their role as possible early manifestations of AF remains insufficiently understood. **Methods**: A systematic review of five relevant studies, comprising a total of 2102 patients, was conducted to evaluate the relationship between vestibular symptoms and AF. **Results**: Dizziness and vertigo were frequently reported, particularly in acute presentations, although they were rarely considered primary symptoms. Evidence suggests a potential correlation between vestibular manifestations and AF, with vertigo possibly acting as an autonomic or inflammatory trigger in selected cases. **Conclusions**: The findings highlight the clinical relevance of including vertigo and dizziness in the routine assessment of patients with AF. Proper recognition of these symptoms may reduce underdiagnosis, improve treatment adherence, and prevent complications associated with misattribution of vertigo to non-cardiac causes.

## 1. Introduction

Atrial fibrillation [AF] is the most prevalent clinical arrhythmia, with a global prevalence of approximately 33 million cases. It has a higher incidence in men, but in women it is associated with increased mortality. The prevalence of AF is rising in parallel with population aging [1,2]. Hypertension, coronary artery disease, and heart failure are the most common comorbidities in patients diagnosed with AF. However, globally, a significant number of cases remain undiagnosed, as AF is frequently asymptomatic. Unfortunately, in such cases, the first clinical manifestation may be its most debilitating complication—ischemic or hemorrhagic stroke.

As the population continues to age, atrial fibrillation has already become a significant medical and socioeconomic concern. Such consequences are responsible for decreased quality of life and considerable economic costs [3].

Clinical symptoms are diverse and may include palpitations, syncope, dizziness, vertigo, or embolic events. Atrial fibrillation may be paroxysmal, persistent, or permanent. [3] Dizziness is a nonspecific symptom that describes a subjective sensation of lightheadedness, vertigo, or imbalance [4]. It may be caused by various factors, including dehydration, low blood pressure, inner ear disorders, certain medications, and central nervous system abnormalities [2,4].

The diagnosis of AF requires a combination of clinical investigations and appropriate treatment of the underlying causes.

Clinical investigations include electrocardiogram [ECG] recordings to confirm the diagnosis, cardiac ultrasound, MRI, CT angiography, and laboratory tests [CBC, blood glucose, etc.] to assess causes, precipitating factors, organ damage, and complications. Diagnosis of AF requires both clinical investigation and appropriate treatment of underlying causes.

## 2. Materials and Methods

### 2.1. Data Sources and Search Strategy

This systematic review was prompted by a clinical observation suggesting a possible correlation between vertigo/dizziness and the onset of atrial fibrillation [AF], which led to the investigation of similar reports in the literature. The data sources included extensive electronic databases: PubMed/MEDLINE, Embase, Scopus, Web of Science, and the Cochrane Library. No language restrictions were applied during the initial search phase. The search was conducted through July 2025 and focused on articles documenting vestibular symptoms [vertigo, dizziness] in the context of atrial fibrillation.

Search terms used “atrial fibrillation” or “AF” and “vertigo” or “dizziness” and “treatment” or “therapy” or “management” and “outcomes” or “compliance” or “quality of life” “ acute vestibular neuronitis”.

The subject of this review is a niche topic with a relatively small number of dedicated articles. We intend to continue following this topic in the future and to carry out a much more comprehensive and registered review at that time.

### 2.2. Study Selection

The selection process was carried out independently by two reviewers. Initially, all citations were screened based on their titles and abstracts. Subsequently, potentially eligible articles were assessed in full text. Any discrepancies were resolved by consensus.

This systematic review was conducted according to the PRISMA guidelines.

The article selection process was represented by a PRISMA flow chart, which illustrates the steps of identification, screening, eligibility, and inclusion of studies.

**Figure 1:**
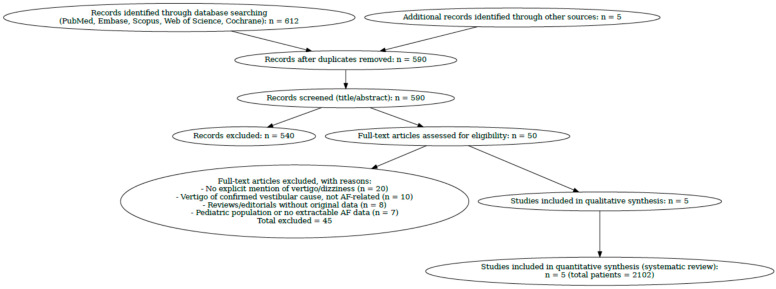
Prisma flow diagram of the study selection process.

The PRISMA diagram illustrates the selection of studies evaluating correlations between vertigo and/or dizziness and atrial fibrillation [Figure 1].

#### 2.2.1. Inclusion Criteria

- Case reports or case series documenting the presence of vertigo or dizziness in relation to atrial fibrillation.
- Observational studies providing relevant symptomatic details [cohort, case–control, cross-sectional], case series, and clinical trials.
- Adult population [over 18 years].
- Articles reporting patients with a confirmed diagnosis of atrial fibrillation.
- Articles evaluating vertigo in the context of AF treatment.

#### 2.2.2. Exclusion Criteria

- Studies without explicit mentions of vertigo/dizziness.
- Articles focusing exclusively on other symptoms [e.g., chest pain, dyspnea].
- Studies including vertigo caused by confirmed vestibular pathologies, unrelated to AF.
- Cases without extractable data on symptomatology.
- Articles published in languages without available translation.
- Reviews, editorials, opinion papers.

#### 2.2.3. Data Extraction

Data extraction was carried out by one reviewer and subsequently verified by a second to ensure accuracy. Articles published in languages other than English were excluded from the extraction process.

Extracted data included:

- Patient sex and age.
- Clinical symptoms at presentation.
- Presence of vertigo or dizziness.
- Prior history of atrial fibrillation.
- Known cardiovascular comorbidities.
- Presence of rapid ventricular response [RVR] [>110 bpm].
- Administered treatment [including oxygen therapy, anticoagulation, etc.].
- Heart rhythm at discharge.
- Vestibular symptoms during follow-up.

## 3. Results

Demographic data, clinical presentations, and therapeutic outcomes in all studies included in analysis were summarized in [Table 1].

### 3.1. Summary of Most Important Patient Characteristics

#### 3.1.1. Demographic Data

In this systematic review five relevant studies were evaluated including a total of 2102 patients with atrial fibrillation. Of these, approximately 1165 [55.4%] were men and 937 [44.6%] women. Patient ages ranged from 32 to 89 years, with women between 34 and 87 years and men between 32 and 89 years. The mean age was 65.5 years in women and 66.7 years in men, with no significant differences. The majority of patients [72%] were over 65 years of age at the time of inclusion. Most patients were treated in an outpatient setting. All included studies confirmed a direct correlation between atrial fibrillation and overweight status, with a reported mean BMI of 31.1 where available. Ethnic origin was predominantly Caucasian [approximately 96%] in the largest analyzed sample [Table 2].

#### 3.1.2. Medical History

In the included studies, none of the patients had a known history of atrial fibrillation at baseline [100%]. The most frequent comorbidities were hypertension [71.7%], diabetes mellitus [28.9%], ischemic stroke [8.6%], and coronary artery disease [16.4%]. In one study investigating the onset of AF in the context of vestibular neuritis [Mirabelli et al., 2023], none of the patients had no cardiovascular history [5].

#### 3.1.3. Clinical Manifestations

Dizziness or vertigo were frequently reported symptoms. In the study by Lin et al., 41.2% of AF patients reported dizziness, and in the study by Mirabelli et al., all patients presented with acute vertigo accompanied by vomiting. Other commonly reported symptoms included fatigue, palpitations, exertional dyspnea, chest discomfort, and nausea. Cluster analysis placed dizziness within the ‘cardiac’ cluster or within the ‘weary’ [asthenic-type] symptom cluster [5,7] [Table 2].

#### 3.1.4. Atrial Fibrillation Profile

Paroxysmal atrial fibrillation was the most frequent form, with a reported prevalence ranging between 51% and 58%. AF episode durations ranged from a few hours to permanent forms. In the acute cases reported by Mirabelli et al., the ventricular response ranged from 100 to 155 bpm. In the study by Ghanbari et al. [11], AF episodes were digitally monitored [ECG HOLTER for 24/48/72 h], and dizziness showed a weak correlation with them [5] [Table 2].

#### 3.1.5. Treatment and Outcomes

Management varied depending on the study. In acute vestibular neuritis cases, treatment included corticosteroids and vestibular suppressants, with spontaneous restoration of sinus rhythm. In larger cohorts, treatments included beta-blockers, antiarrhythmics, calcium channel blockers, and anticoagulants. Radiofrequency ablation was performed in up to 52% of patients [Bang & Park, 2023] [8]. Overall, dizziness and vertigo improved either with restoration of sinus rhythm [60.7%] or following treatment of the underlying vestibular cause [27.2%] [Table 2].

## 4. Discussion

This systematic review highlights a frequently underappreciated clinical dimension of atrial fibrillation [AF]: vertigo and dizziness as significant symptoms in the presentation and progression of the disease. While palpitations, dyspnea, and fatigue are typically considered hallmark AF symptoms, the included studies suggest that dizziness and vertigo may occur in isolation or complete certain symptom clusters. These may significantly influence patients’ perception of the disease and therapeutic decisions. However, the body of evidence reviewed here suggests that vestibular symptoms, although often considered secondary, may carry substantial clinical significance.

From a pathophysiological perspective, the association between AF and vestibular dysfunction may be mediated by autonomic nervous system imbalance. Huang et al. (2023) highlighted the importance of autonomic pathways in AF initiation and perpetuation [2]. Acute vestibular neuritis, as reported by Mirabelli et al. (2023) [5], may act as an autonomic trigger, producing inflammatory and neural changes that predispose to AF onset. This observation shifts vertigo from being merely a coincidental symptom to a potential upstream mechanism of arrhythmogenesis. Future studies should explore inflammatory biomarkers and autonomic indices (e.g., heart rate variability, vagal tone) in AF patients presenting with acute vertigo to better understand causal relationships.

The study by Mirabelli et al. reported three acute cases of severe vertigo associated with vestibular neuritis, which preceded or coincided with the onset of atrial fibrillation. These patients had no cardiovascular history, suggesting that acute vestibular dysfunction might trigger an autonomic or inflammatory response contributing to the onset of AF. This offers an interesting etiopathogenic perspective, proposing vertigo not only as an associated symptom but also as a possible precipitating or even triggering factor for AF [5].

In large-sample studies such as Lin et al. (2024) [7] and Bang & Park (2023) [8], dizziness was reported in up to 41% of patients, though rarely as the primary symptom. Instead, it appeared integrated within “cardiac”-type symptom clusters, alongside chest discomfort and palpitations, or the “weary”-type, associated with fatigue, suggesting an overlap between cardiac dysfunction and the subjective perception of exhaustion or instability. These findings reinforce that dizziness should not be evaluated in isolation but rather within the broader context of symptom networks. Importantly, Bang & Park demonstrated that patients reporting dizziness had significantly lower quality-of-life scores and higher psychological distress, underscoring the functional burden of these symptoms.

Another notable aspect is that in the study by Streur et al. (2017), dizziness correlated with older age and poor physical function, being significantly more frequent in patients with multiple comorbidities, raising concerns about underdiagnosis in vulnerable populations [9,10].

Although dizziness was not identified as a decisive factor in choosing therapeutic strategies [rate vs. rhythm control], the studies suggest that the presence of vertigo or dizziness may influence treatment tolerability or the patient’s perception of the need for intervention. In the study by Bang & Park, dizziness was associated with increased psychological distress and lower quality-of-life scores, suggesting an indirect influence on treatment compliance [8].

Moreover, in the acute cases reported by Mirabelli et al., improvement of vertigo was closely linked to the restoration of sinus rhythm, reinforcing the hypothesis of a functional or reversible autonomic mechanism sensitive to AF treatment [5].

Sex- and age-related differences represent another critical dimension. Blum et al. (2017) [12] and Daneshvar et al. (2018) [13] documented that women are more likely to report atypical symptoms, including dizziness, compared with men. Moreover, in elderly patients, dizziness is often attributed to non-cardiac causes such as vestibular degeneration or polypharmacy, increasing the risk of underdiagnosis [14,15]. Our synthesis suggests that dizziness in women and older adults with AF may not only reflect comorbidity but could represent a sentinel symptom. This necessitates sex-specific and age-sensitive diagnostic frameworks.

This notable aspect is also found in the study by Streur et al. (2017), dizziness correlated with older age and poor physical function, being significantly more frequent in patients with multiple comorbidities, raising concerns about underdiagnosis in vulnerable populations [9,10].

Another key clinical concern is diagnostic overlap between vestibular vertigo and cerebrovascular events. Chang et al. (2024) [4] demonstrated that acute dizziness in emergency settings carries a significant risk of stroke, particularly posterior circulation infarcts. In AF patients, where thromboembolic risk is intrinsically elevated, overlooking vertigo as a potential ischemic warning sign could delay life-saving interventions. Similarly, amaurosis fugax (Kaur et al., 2025 [16]) may be misinterpreted as a vestibular disturbance, further complicating differential diagnosis. These findings stress the urgent need to refine triage and diagnostic tools that integrate vertigo into AF risk assessment.

A critical issue identified is the systematic omission of vertigo and dizziness from standard AF symptom evaluation tools (e.g., AFSymp™) [17]. This may lead to the oversight of atypical or subtle presentations, especially in elderly or female patients, where vertigo may be the only significant clinical sign. In patients hospitalized for heart failure complicated by AF, dizziness was more frequently reported by women [12]. This suggests the need for careful symptom assessment, particularly in female patients where vertigo may be the primary clinical manifestation [13]. Current symptom assessment instruments, such as the AFSymp™, do not include dizziness or vertigo as independent items [17]. This omission contributes to systematic underreporting in both research and clinical practice. Including vestibular symptoms in validated AF-specific questionnaires may improve clinical recognition, research comparability, and ultimately patient outcomes. In addition, digital health solutions such as mobile applications have shown promise in capturing real-time reports of dizziness alongside other AF symptoms, providing a more comprehensive picture of disease burden [11].

Therefore, management of AF in older adults is often complicated by atypical presentations, including dizziness which was often underappreciated. Due to age-related changes and comorbidities, vertigo or imbalance may be underreported or misattributed, leading to diagnostic delays [14,15].

Vertigo in AF patients should be carefully distinguished from other transient neurological symptoms. Amaurosis fugax, a sudden transient monocular vision loss due to retinal ischemia, can occur in patients with AF and may be mistakenly interpreted as a vestibular symptom [16]. Exercise-induced atrial fibrillation [AF] can present with symptoms like dizziness or vertigo, likely due to abrupt hemodynamic changes and autonomic imbalance. A recent case report described AF triggered by exertion, with dizziness being one of the initial symptoms observed [18]. Therapeutic interventions for AF, such as atrial antitachycardia pacing, have also been reported to provoke dizziness. This may be due to brief alterations in cardiac output or cerebral perfusion during pacing episodes [6].

The development of mobile applications for AF symptom monitoring has shown promising results. Dizziness was among the commonly tracked symptoms, enabling real-time feedback and better personalization of care [11].

Finally, therapeutic implications deserve emphasis. While rate or rhythm control strategies are generally guided by traditional symptoms, the presence of dizziness may influence treatment adherence and patient willingness to pursue invasive procedures. For instance, in Bang & Park’s study [8], dizziness, as stated above, correlated with higher anxiety levels, which may indirectly affect compliance with anticoagulation or ablation. Moreover, dizziness occurring during atrial antitachycardia pacing highlights the need to balance therapeutic efficacy against tolerability [6].

These findings suggest that vestibular symptoms should be integrated into both risk stratification and shared decision-making processes.

## 5. Conclusions

There are few clinical studies in the literature addressing the prevalence of vertigo in the context of cardiovascular diseases. The literature is limited to several case reports without detailed clinical characteristics or diagnostic criteria.

The findings of this review highlight that vertigo and dizziness, although often underrecognized, represent clinically significant manifestations in atrial fibrillation. Their presence may signal underlying autonomic dysregulation, inflammatory triggers, or cerebrovascular risk, and may substantially affect patients’ quality of life. Despite these implications, the current literature remains sparse, fragmented, and largely descriptive.

Dizziness and vertigo are challenging clinical manifestations requiring multidisciplinary involvement, including neurology, cardiology, otorhinolaryngology [ENT], and even psychiatry. Life with chronic dizziness is burdensome for affected individuals, and the literature recognizes the diagnostic and functional implications chronic dizziness entails.

Dizziness may often become chronic due to difficulties in obtaining a clear diagnosis and implementing effective treatment.

The etiological factors of dizziness involve both the neurological and cardiovascular systems, making differential diagnosis complex.

If vertigo occurs as a result of vestibular neuritis, treatment may include corticosteroids or other medications to reduce inflammation and manage symptoms. However, their use is limited due to potential side effects and the increased haemorrhagic risk in AF patients who require oral anticoagulant therapy, particularly with long-term use.

Vagal nerve stimulation is a recently introduced treatment for AF that involves stimulating the dominant auricular branch of the vagus nerve located in the external auditory canal and the skin of the auricle. It has been effective in reducing the incidence of AF.

If vertigo is associated with stroke, treatment may involve thrombolysis, antiplatelet and anticoagulant therapy, or other interventions to improve cerebral blood flow. Antiplatelets and anticoagulants are safe and effective in treating vascular-related vertigo and dizziness by preventing new ischemic events and increasing posterior circulation flow, thereby reducing episodes of vertigo/dizziness and imbalance disorders.

The late diagnosis of atrial fibrillation or the underlying cause of vertigo can lead to prolonged symptoms, potential complications, and increased risk of adverse outcomes.

It is essential to distinguish isolated vascular vertigo/dizziness from non-vascular disorders, such as acute unilateral vestibulopathy/vestibular neuritis involving the labyrinth or vestibular nerve, as treatment strategies and prognosis differ between these conditions.

Benign paroxysmal positional vertigo is a common cause of peripheral vertigo. It can be easily diagnosed using the Dix–Hallpike maneuver and treated with repositioning maneuvers. Recurrent vertigo with tinnitus indicates Ménière’s disease, and low-frequency hearing loss can be easily documented via audiometry.

Distinguishing between dizziness and vertigo caused by posterior circulation stroke and vestibular disorders can be truly challenging. A thorough history regarding the timing and triggers of these symptoms, combined with bedside clinical tools, including the Dix–Hallpike maneuver, assessment of ocular movements, hearing evaluation, and neurological examination, can aid in diagnosis.

Misdiagnosing acute stroke may result in missed opportunities for effective treatment, increasing morbidity and mortality, while overdiagnosing vascular vertigo/dizziness could lead to unnecessary and costly investigations and medication.

From this analysis, we found out that several important gaps emerge: 1. prospective multicenter studies are urgently needed to quantify the prevalence and prognostic significance of vertigo in AF, moving beyond anecdotal case reports; 2. mechanistic research should investigate autonomic and inflammatory biomarkers in patients presenting with vestibular symptoms, exploring causal pathways between vestibular insults and AF onset; 3. sex- and age-specific analyses are required to clarify whether dizziness carries different predictive value in women and elderly populations; 4. The refinement of clinical tools, including revision of AFSymp™ and development of digital monitoring applications, should incorporate dizziness and vertigo as standard symptom endpoints; 5. stroke prevention strategies should integrate vertigo as a potential red flag for cerebrovascular ischemia in AF patients, ensuring timely neuroimaging and therapeutic interventions.

Addressing these gaps has both scientific and clinical importance. From a patient perspective, recognizing and validating vertigo as part of the AF symptom spectrum could reduce diagnostic delays, improve treatment adherence, and enhance quality of life. From a health systems perspective, better symptom characterization may reduce misdiagnoses, avoid unnecessary investigations, and ultimately decrease the burden of AF-related complications such as stroke.

In conclusion, vertigo and dizziness should no longer be viewed as peripheral or coincidental complaints in AF. Instead, they represent a meaningful dimension of disease expression and patient experience. It is essential to accurately identify the cause of vertigo to ensure appropriate treatment and prevent potential complications arising from both AF and other associated causes of vertigo. Systematic recognition, standardized assessment, and targeted research are the next steps toward improving outcomes for this large and vulnerable patient population. In this way, the quality of life for patients will continue to improve through better healthcare interventions for AF patients, reducing depression and anxiety.

We identify the need for further studies to investigate the relationship between vertigo and arrhythmias.

The protocol of this systematic review was not recorded in a public registry.

## Figures and Tables

**Table 1:** Demographic data, clinical presentations, and therapeutic outcomes.

Study	Total Patients	AF Diagnosed	Mean Age (yrs)	AF Type	% With Vertigo/Dizziness	Vertigo Diagnosis
Mirabelli et al. [5]	1	1	68	paroxysmal	100	Vestibula neuritis
Gianni et al. [6]	1	1	72	Pacing-induced	100	Pacing symptoms
Lin et al. [7]	201	65	52–83	41.2	Network symptoms	Beta-blockers, anticoagulants
Bang & Park [8]	198	64.2	40–73	35.3	Cluster	diversified
Streur et al. [9,10]	1701	63.5	32–89	29.8	Secondary symptom	diversified

**Table 2:** Summary characteristics of patients included in the analysis.

Category	Value [with Percentage]
Total patients	2102 [100%]
Male patients	1165 [55.4%]
Female patients	937 [44.6%]
Average age + range [males]	66.7 y [32–89]
Average age + range [females]	65.5 y [34–87]
Hypertension [HTN]	1507 [71.7%]
Diabetes mellitus	607 [28.9%]
Stroke	180 [8.6%]
Coronary artery disease	344 [16.4%]
Improved after rhythm restoration	250 [60.7%]
Improved after vestibular treatment	112 [27.2%]

## Abbreviations

The following abbreviations are used in this manuscript:

AF	Atrial Fibrillation
